# Supplementary material for: What matters when doctors die: A qualitative study of family perspectives
Source: PLoS One. 2020 Jun 23;15(6):e0235138. doi: 10.1371/journal.pone.0235138 (PMC7310709; doi:10.1371/journal.pone.0235138)
Supplement: S1 Appendix — Additional quotes from interviews of family members of physicians in the Johns Hopkins Precursors Study presented by theme. (DOCX) [file pone.0235138.s001.docx]

**S1 Appendix.** **What Matters to their Families When Doctors Die?** Additional quotes from interviews of family members of physicians in the Johns Hopkins Precursors Study presented by theme.

| **Honoring preferences for context of end of life care** |
| --- |
| **Who is a caregiver and the location of care** |
| Well, just for the fact that he was there and not home, because he was always deathly afraid that he would end up in a nursing home, for example, and bear in mind that he was used to making hospital rounds when he was practicing medicine and also nursing home rounds, and he did not want to be a part of that. I mean, he did not want to be in a nursing home or in the hospital, because he felt constricted. Family Member 2094 |
| I think she was pretty extraordinary all the way through because she was so clear about it and was so helpful for all of us. I was definitely afraid I’d have to make that decision [initiating hospice care] for her when she wasn’t capable of it. And she was lucid enough and clear enough that I didn’t have to make the call. I just had to support her…There were lots of challenges and so forth but she was so glad to be home. And she had three or four really lucid days. She got to visit with her sister and other friends and family. Family Member 5648 |
| He loved his home. We were there for 42 years and he loved it and we-- yeah. I was able to hire some help during the day in that last year and yeah. So he was right at home…. And so, we kept him right in the middle of the family room. We were all with him and we read to him and we played music, his favorite music, and he was surrounded by his paintings and his family and it was a good death. Family member 5868 |
| **Team ‘care’** |
| I was his chief nurse. I made sure that he got his--made sure he got his medications every day. I contacted his doctors when I thought he needed some sort of assistance… and we have children in the area who were very attentive. We really had sort of a family program. I have a son who's a doctor, married to a doctor and they live 6 blocks away. Family member 5113 |
| He elected to include my brother and me and we were happy to help him. It was just not something he wanted to do by himself. And we were all on the same page, for sure. And we had good communication about it and we were all able to reach what we felt like was the best decision we could make at the time. Family member 8365 |
| It was this whole group-- this group effort, and having my sons here and my daughter-- just having the family support me made me feel that I was doing the right thing, and his cousin the nurse too. It just was a very cohesive, supporting environment. I don't think I would have had that at the hospital. Family member 4536 |
| Well, I had of course my sister and my brother, and my brother was great on the medical stuff, but he's not great on emotional stuff, so my sister was more of that person, but it was generally a very lonely feeling, because I had moved. I didn't know anybody where we were. Family member 8495 |
| **Challenges within the healthcare system** |
| And I was very comfortable with our internist and neurologist, and again the intern had been a student. The internist has been a student of <SPOUSE NAME>'s. And so, again, very comfortable relationships. I think physicians are rather lucky in that way. Yeah, I mean because they often know the people personally and it makes communications very, very easy. Family member 5868 |
| I guess he was in the intensive care overnight and the next day I guess he called me at six o'clock in the morning. I ran back up to the hospital. I'd gone home at one, and he said "Come back up to the hospital." When I got there I guess I gave him a kiss and held his hand, and he died right there, and then the docs came running in and said "Oh, we want to do CPR," and I said "Nope." I had already given them the papers, and I said "No, he doesn't want anything like that, and that's not to be done," so that was the end of that. Family member 5795 |
| She's a sick, and of course I didn't think they were giving her aggressive enough care that could have prevented all those aggressive measures, but no, it was very hard to kind of take this DNR, standing DNR, no heroics measure, and then have to break it down to an individual event. I just-- it's hard, even as a physician, to understand what is the right decision in that matter. Family member 3265 |
| He was particularly concerned about young physicians who hadn't had a lot of experience and may not have a lot of wisdom. He didn't want them limiting the amount of morphine because it “might” suppress his respirations. Family member 6431 |
| Yes, I think so, and also the last hospitalization-- I don't think the last young doctor who treated him knew that he was a doctor himself, and he just-- it was a negative experience. His attitude and demeanor was like, "Well, you just take this"-- he was having some bowel-- he had had some bowel obstruction, which I'm not sure was actually related to the cancer or not, but it was a significant problem, and when he went back in the very last time, they just wanted to give him the kind of-- they gave him like the empty container with the salts in it that you use to prepare for a colonoscopy. Well, he was in no condition to be doing something like that. So, "Take this home, because we can't get anything moving here," and it was just before Christmas and they always say it's not a good time to be admitted into the hospital during the hospital season because the staff might be reduced. Family member 4536 |
| **Supporting the patient to preserve control and dignity in care** |
| **Control** |
| I remember there was no question that heroics of any kind were not appropriate or even treatments. I don't think he objected to that and I think everybody was very comfortable that nothing should be done…I mean he wasn't up-to-date maybe on all of the treatments that were available to him and the nurse even said "Is he always this bossy?" <laughter> And I said "Yes, and sometimes he's even worse." So he just felt more comfortable. Sort of a control freak if you want to call it that. I remember there was no question that heroes of any kind were not appropriate or even treatments. I don't think he objected to that and I think everybody was very comfortable that nothing should be done. Family member 3265 |
| Yeah, I think-- I think he was aware that mentally he was failing. Because what shocked me when-- …I was there when he handed me his checkbook and said, “You have to take care of this, I can’t do it anymore.” So, that was when I realized he really had slipped. Family member 4821 |
| So he very much took care of all of his medical needs. I did not know what pills he took. He insisted on, most of the time, going to chemo by himself or taking himself. He was very independent about anything to do with his health, because he knew I didn’t know anything about it. And so I never said, “Well, I don’t think you should do this or not do that,” because I didn’t know. Family member 4085 |
| **Maintaining dignity despite functional or cognitive decline** |
| …at night he'd try to get up to go to the bathroom or something like that, and I would help him, but he tried to do-- it was that independence. He tried to do a lot of things himself. It was just difficult for him to, and so it was much better for somebody to be there to help him. Family member 8755 |
| I tried to influence him to get a bit more exercise, walk out in the backyard, do that kind of thing but he pretty much had his own way and wasn't too adapted. Family member 6062 |
| "Well, you know how it is up there. It's gigantic, where you park, if you come late," and he was already having some trouble walking, and so I said, "Please let me come and just sit at the back. I'll just take my iPad and no one will even know I'm there." So I did that, and no one knew how sick he was. Family member 4536 |
| **Maintaining dignity through goal-setting** |
| Well, he knew in any case that it would be terminal, but he wanted to extend his life, hoped to extend his life long enough so that he could live to see those two things, his granddaughter graduating from medical school and the library finished, and so he was willing to try the oral medication with that end in mind. Family member 2094 |
| **Developing a shared understanding** |
| **Discussions about care were casual and sparse** |
| I found myself in some pretty strange places and having to make some decisions that I didn't really want to make. Family member 9536 |
| She and my father both prepared a trust and part of that also included some instructions. We also had a couple of family meetings about their wishes. In [STATE], of course, we have the POLST, the physician’s order for life saving treatment, and she had one of those as well. Family member 5648 |
| **Finding common ground** |
| But, you know, he basically said, “If I can still bat an eyelash and everything else is gone and I’m still there somewhere I want you to do everything you can.” And my mom was in a very different place which was, “I’ve lived a great live. I want you to do X, Y and Z. But I’m not there. I don’t want that for me.” So having that contrast, and having that express conversation with both of them was incredibly helpful because it helped sort of put the continuum in play so we could, me and my brother, could understand really clearly where they both were. Family member 5648 |
| I think a planning session that the conversation relieved my mind a great deal because I think the worry of the caretaker is that something will happen to you, that I'll fall and break a hip or have an auto accident or something suddenly and then you suddenly have to turn everything over to someone else. So I think having a written plan really took a lot of pressure off of me because I knew that the kids all had copies and they would know what their father wanted. So to me-- so it was the keeping life normal, as normal as possible, having a plan, and then help at the end. I think those were sort of my three starred items. Family member 5868 |
| I just sit around and wonder about people who don’t have anyone who really is just looking after their best interest. They're just at the mercy of a system. It just makes me wonder just-- even if everything's being done properly, if there's just no-- it seems to me, in our case, there was no sort of substitute for that advocacy. Family member 9415 |
| **Coping with the aftermath of bereavement and regrets** |
| So I had a bit of guilt because we sort of encouraged him. Being a surgeon he sort of didn't-- he knew the downsides of surgery. And so, he hesitated and we hoped it would help him but it didn't at all. It set him back, but you can't live backwards. Family member 5868 |
| I wanted him to go to the doctor a week before he died but he just wouldn't do it and I should've taken him but I was afraid to try and get him to follow. He would get very upset if I'm trying to push him to do that and I didn't want his blood pressure to go up because he did have-- he was on blood pressure medicine. Family member 5934 |
