# Supplementary material for: What matters when doctors die: A qualitative study of family perspectives
Source: PLoS One. 2020 Jun 23;15(6):e0235138. doi: 10.1371/journal.pone.0235138 (PMC7310709; doi:10.1371/journal.pone.0235138)
Supplement: Attachment — Submitted filename: Response to Reviewers .docx [file pone.0235138.s003.docx]

We are truly grateful for the high-quality critique provided by the reviewers who were incredibly thoughtful in their suggestions. We have addressed all comments from the editor and reviewers in the tables below and believe that the manuscript has improved clarity and focus due to these changes.

| **Editor’s Review** | |
| --- | --- |
| Critique | Response |
| Both reviewers made important comments regarding the focus of the manuscript. I concur with the reviewers as you have provided readers with conflicting messages regarding the aims of this study. On P. 4, lines 74-77, you stated that “In essence, we wanted to know, “What matters most to their families when physicians die?” The purpose of this study was to identify common challenges and strategies used by families in end-of-life decision making in a situation where the patient, a physician, had an excellent understanding of treatment options and prognosis.” However, on P. 5, lines 88-89, it was stated that “present study was designed to examine physician preferences for end-of-life care and their approach to decision making”. Please, state clearly all aims of the study and confirm that analyses are consistent with those aims throughout the manuscript. | We agree that there was a lack of clarity about the focus of the manuscript. We have clarified the focus of the manuscript within the context of the broader end of life study and how that study fit into the Precursors cohort study in the design section Pg. 5-6 |
| Although you have stated that “Study procedures including informed consent are annually reviewed and approved by the Johns Hopkins University School of  Medicine Institutional Review Board (IRB00051731)”, that description by itself does not guarantee that the subjects that were interviewed provided informed consent. | Family members and physician participants were contacted by letter, and oral consent was provided before proceeding with the interview. We have included these details in the sample selection section Pg 7. |
| Both reviewers requested more information about patients and interviewees. Please consider presenting a table with the characteristics of patients (e.g. gender, age [mean and standard deviation or median and interquartile range], main diagnostic leading to the patient’s death, medical specialty, classification of end-of-life preferences) and another table with the characteristics of the family members who were interviewed (e.g. gender, age, profession, type of relationship with the patient, whether the interviewee was the primary healthcare representative of the patient or not). | Unfortunately, we collected a very small amount of information about the family members, which we have provided. We have also added a note in the limitations paragraph. |
| Finally, I noticed that several items of the COREQ (Consolidated criteria for REporting Qualitative research) (<http://intqhc.oxfordjournals.org/content/19/6/349.long>) were not addressed in this report. For instance, there is no information about how participants were selected in terms of inclusion and exclusion criteria. Please, revise the manuscript accordingly and provide a COREQ checklist indicating the page numbers where each item was addressed as supplementary material. This is a very important issue that must be addressed by the authors since adherence to appropriate reporting guidelines is one of the publication criteria adopted by PLOS ONE (<https://journals.plos.org/plosone/s/criteria-for-publication>). | We have provided the additional details required by the COREQ checklist and submitted the checklist with the supplementary materials. |

| **Journal Requirements:** | |
| --- | --- |
| Item to address | Response |
| Please ensure that your manuscript meets PLOS ONE's style requirements, including those for file naming. The PLOS ONE style templates can be found at  <http://www.journals.plos.org/plosone/s/file?id=wjVg/PLOSOne_formatting_sample_main_body.pdf> and <http://www.journals.plos.org/plosone/s/file?id=ba62/PLOSOne_formatting_sample_title_authors_affiliations.pdf> | Changed file names |
| Please modify the title to ensure that it is meeting PLOS’ guidelines (<https://journals.plos.org/plosone/s/submission-guidelines#loc-title>). In particular, the title should be "specific, descriptive, concise, and comprehensible to readers outside the field" and in this case the title may not convey sufficient description of the methods or analysis undertaken. | Changed title to What matters when doctors die: A qualitative study of family perspectives |
| We note that you have stated that you will provide repository information for your data at acceptance. Should your manuscript be accepted for publication, we will hold it until you provide the relevant accession numbers or DOIs necessary to access your data. If you wish to make changes to your Data Availability statement, please describe these changes in your cover letter and we will update your Data Availability statement to reflect the information you provide. | Relevant excerpts from our transcripts are provided in our text and tables. We have therefore changed our data availability statement. |

| Reviewer Critique | Response |
| --- | --- |
| Reviewer 1 | |
| **Introduction** - "From the point of view of families, quality of end-of-life care decreased from 2000 to 2013 despite the many efforts to improve services provided at the end of life." Where was this assessed? As this is an international journal, it would be adequate that such affirmations were contextualised in more detail as end-of-life care quality varies hugely among and within countries. | We have provided the context for the US-based reference and added some global context for the problem as well. Background Pg 4 |
| - "Poor quality of care at end of life impacts families even after the death of a loved one, putting them at risk for prolonged grief." I would like to see information about the importance of good quality end-of-life care for the people who are at the end of their lives too or about the public health need for improvement in end-of-life care more broadly. | We have added information related to the global public health need for improved quality of end-of-life care and the implications for patients as well in the Background section Pg 4 |
| - "Many studies have identified that patients often have a poor understanding of prognosis and treatment options preventing high quality end of life care." This sentence sounds dubious - please rewrite this for clarity. | We have deleted this sentence. |
| **Design** - "As this cohort advanced in age, end-of-life issues became more important; the present study was designed to examine physician preferences for end-of-life care and their approach to decision making." I understand the study was about the families’ views about what matters at the end-of-life of patient-physicians, and not explore the patient-physicians’ preferences? Please clarify this. | We have re-worked the design section to clarify this point. Pg 5-6 |
| - I would like to see more details about the methodology used and rationale for it: I understand constant comparison to be more of an inductive qualitative technique or method for data analysis, rather than a study methodology in itself. Also, normally, constant comparison method is jointly used with theoretical sampling, which was not the case in this particular study. Please provide more information/justification on these aspects. | We agree the constant comparison method is really at its core grounded theory, an inductive approach to data analysis, which we used jointly with some deductive coding. We did not view this as a study methodology. We used constant comparison in conjunction with theoretical sampling based on the class of treatment preferences. With improved clarity of the purpose, design and sampling, we believe this point has been addressed. |
| - I think it would be important for the reader to contextualise the findings if the diagnoses of the patient-physicians were provided (cancer; dementia; etc). | We did not engage in diagnosis discussions, although many family members shared details of the physician’s course of illness and death. |
| Results - Page 8: "As described elsewhere, the cohort had varied attitudes towards aggressive treatment, despite their advanced age." - why would one expect attitudes towards end-of-life treatment vary with age? | We removed ‘despite their advanced age’. |
| - Appendix: I think the quotes within the themes “Challenges with the healthcare system” and “Control over symptoms” do not quite represent its titles and therefore these need careful revision. For example, the first quote in “Challenges with the healthcare system” seems to be about the particular benefit of being a physician when it comes to end-of-life preferences and care as there is better communication and respect of patient preferences. Within “Control over symptoms”, the first sentence clearly seems to be about acceptance; and the second and third quotes which seem to be more about having control over one’s overall life and decisions, rather than control over symptoms. | Thank you for this suggestion. We have provided these sub-themes/codes to help provide some structure to the table – similar to a coding tree. The first quote is an exemplar of a divergent quote which we wanted to fairly represent.  We have modified the heading “Control over symptoms” to just “Control”. Pg 10-13 |
| Discussion Typos: lines 333 and 351 Line 345: “life-threatening illness” – do you mean life-limiting illness? | We apologize for these typos and have addressed them. We also changed line 345 to life-limiting illness. |
| Reviewer 2 | |
| My main comment is that of article focus. While the title and part of the manuscript place the focus on informal care giver experience, both the topics for the semi-structured interview and majority of your results focus on what mattered to the now-deceased practitioners - even if by proxy. Caregiver own needs seem less of a focus in the way you present the results, too.  Nonetheless, I appreciate that post-bereavement interviews with informal carers are likely to involve carer focus on the needs of the patient even if the interview questions emphasise carer needs and experiences. Literature on conceptualising carers and service users/patients as dyads, which acknowledges the complex interdependencies of need may be useful here. Overall, the focus of the manuscript should be clearer and consistent throughout - whichever way you chose to approach this. | We agree with this point as noted above. We have re-written the purpose statement and design section to improve clarity. Although incredibly important, caregivers’ needs were not the focus of this study. |
| I understand that the wordcount is not limitless, but currently the background section is rather brief. Clearer situating of your research in wider literature on the topic would be beneficial.  Studies specifically on healthcare professional own death and end of life care are not abundant, but they do exist (e.g. Gray CH. How will your wife cope when you die? Doctors' widows supply some answers. Canadian Medical Association Journal. 1980 Jan 26;122(2):206.).  What matters to families and care givers is covered fairly extensively and again can help you situate your study. This would in turn help to further clarify to the reader why your research matters. You are right in saying that a physician-patient cohort have considerably more healthcare literacy and understanding of illness trajectories than other patient groups, and that therefore they are better-able to make informed advance decisions - but tell the reader why this matters? Is it, for example, about looking at what issues are more universal across patient populations? Is it about greater caregiver well-being, if carers are spared some complex decision making? You do provide a purpose for your study, but explaining 'why' that is the purpose would help your reader. | We have added a section describing known factors that influence a good death, or what matters to families and patients at end of life. Thank you for the reference; we have included it as well. Pg 4  Pg 5, Paragraph 3 of the background section is re-worked to explain why the physician/family perspective matters. |
| In the discussion section, your manuscript would benefit from a clearer flow of the overall argument. At the moment the discussion is slightly disjointed and the many implications you mention could be connected in a more coherent manner. This may be because - as mentioned earlier - the focus shifts between informal caregivers and physicians at the end of life... as well as between recommendations for practice and specific focus on dignity. What is the one main point you would like the reader to take away from this article? What is the main contribution that you are making to the body of literature on the topic? A clearer structure, directionally building to that main point, would help the reader follow the arguments. | We have carefully read and re-read the discussion, and made some changes in the sequence that we hope will improve the flow. |
| Below are some smaller points: - Although your interviews touched on divergence of family member wishes and approaches, overall you spoke to (primary) informal caregivers. I am therefore wondering if reference to family wishes and priorities (i.e. implying the whole family) is somewhat misleading. | We used ‘family’ to be inclusive of spouses, siblings and children. We have clarified this point in the methods Pg 6 and added this point to the limitations. Pg 20 |
| - You acknowledge from the start that physicians are a 'unique' patient population, but so are their informal caregivers. You touch on this in the discussion, but could also benefit from briefly discussing this when defining the sample. Caregivers, too, may have a greater than average health literacy, be practicing or retired healthcare staff themselves, be more affluent than the general population and afford better care, etc. Some of this may also be important in the limitations section. | We have addressed this point in the limitations as suggested. Pg 19-20 |
| - PLoS One has an international readership and it would be helpful if you specified the context when relevant (e.g. lines 61-62 refer to the US). Similarly, some country-specific terminology - such as 'Medical Directive' - would benefit from a very brief definition or addition of alternative terms used elsewhere (e.g. 'living will'). | We have provided context for our references in the background. Pg 4  The Medical Directive is provided in the reference 19, nevertheless, we have included additional information on the instrument. Pg. 6 |
| - It would be interesting to know the proportions of participants who were caring for physicians with 'most aggressive', 'intermediate' and 'least aggressive' care preferences. | We have added this information to our table. Pg 9 |
| - In the participant table you break down adult children by gender, but do not do so for spouses. A uniform approach in either direction is advisable. | We have corrected this – in this case there were no husbands, only wives of deceased physicians. Pg 8 |
| - It is more conventional to describe study participants in the methods section (e.g. see Christenson A, Johansson E, Reynisdottir S, Torgerson J, Hemmingsson E. Women's perceived reasons for their excessive postpartum weight retention: a qualitative interview study. PLoS One. 2016;11(12)). Similarly, adding participant codes or pseudonyms to the quotes you use in the result section is both conventional and helps the reader understand if you are over-relying on any one transcript or if quotes used reflect all/most of your participants. | We have replaced all participant codes for all quotes in the text.  We found several exemplars published in this journal with sample description in the results section, consistent with other papers we have published, but we would be glad to make changes if instructed by the editor. |
| - Line 321 - it could be clearer whom you are referring to by using the term 'providers' | We clarified we meant healthcare providers Pg 20 |
| - Lines 338-339 - please add a reference. | For the statement, “Physicians were, however…” on lines 338-339, the reference is the same as the previous sentence. |
